# Integrating CT‐Based Radiomics Analysis With Serum CEA Level to Predict Neoadjuvant Chemoradiotherapy Response in Colorectal Cancer

**DOI:** 10.1002/kjm2.70241

**Published:** 2026-05-26

**Authors:** Sin‐Hua Moi, Ming‐Yii Huang, Shu‐Han Yang, Chien‐Jang Chen, Ying‐Pei Jhong, Tun‐Wei Hsu, Chien‐Chih Ke

**Affiliations:** ^1^ Graduate Institute of Clinical Medicine, College of Medicine Kaohsiung Medical University Kaohsiung Taiwan; ^2^ Faculty of Medicine, College of Medicine Kaohsiung Medical University Kaohsiung Taiwan; ^3^ Department of Medical Research Kaohsiung Medical University Hospital Kaohsiung Taiwan; ^4^ Center of Applied Genomics Kaohsiung Medical University Kaohsiung Taiwan; ^5^ Precision Sports Medicine and Health Promotion Center Kaohsiung Medical University Kaohsiung Taiwan; ^6^ Research Center for Precision Environmental Medicine Kaohsiung Medical University Kaohsiung Taiwan; ^7^ Department of Radiation Oncology, Kaohsiung Medical University Hospital Kaohsiung Medical University Kaohsiung Taiwan; ^8^ Department of Radiation Oncology, School of Medicine, College of Medicine Kaohsiung Medical University Kaohsiung Taiwan; ^9^ Center for Cancer Research Kaohsiung Medical University Kaohsiung Taiwan; ^10^ Department of Medical Imaging and Radiological Sciences Kaohsiung Medical University Kaohsiung Taiwan; ^11^ Department of Radiology Taipei Veterans General Hospital Taipei Taiwan; ^12^ Drug Development and Value Creation Research Center Kaohsiung Medical University Kaohsiung Taiwan

**Keywords:** carcinoembryonic antigen, colorectal cancer, CT‐based radiomics, neoadjuvant chemoradiotherapy, pathological complete response

## Abstract

Response to neoadjuvant chemoradiotherapy (NACRT) in locally advanced colorectal cancer (CRC) varies widely, and accurately identifying poor responders is crucial for guiding timely treatment modification. This study aimed to develop an integrated predictive model combining radiomics derived from routine radiotherapy non‐contrast planning computed tomography (CT) images with serum biomarkers to achieve better predictive accuracy than modality alone in identifying the therapeutic response of CRC patients receiving NACRT. Ninety‐two patients with stages II–III CRC who received NACRT and surgery were retrospectively analyzed. CT‐based radiomic features were extracted from pre‐treatment non‐contrast planning CT images using LIFEx software. Patients were randomly divided into derivation (*n* = 65) and validation (*n* = 27) cohorts while least absolute shrinkage and selection operator logistic regression was used to construct a radiomics score (Rad‐score). Univariate and multivariate analyses evaluated the independent predictive value of Rad‐score and patients' clinicopathological features. A nomogram was established and calibrated using bootstrap resampling, and of 111 usable radiomic features, seven were selected to construct the Rad‐score. The CT‐based radiomics signature showed strong discriminative performance in predicting pathological complete response (pCR) (AUC: derivation 0.854; validation 0.818). In multivariate analysis, Rad‐score (adjusted OR = 10.20, *p* = 0.002) and low pretreatment serum carcinoembryonic antigen (CEA) level (adjusted OR = 15.20, *p* = 0.024) were independent predictors of pCR. The integrated nomogram demonstrated excellent calibration in both cohorts [mean absolute error (MAE) = 0.042 and 0.045]. A combined model incorporating CT‐based radiomics and pretreatment serum CEA provides a robust and resource‐efficient tool for predicting therapeutic response following NACRT in CRC.

## 1 Introduction

Colorectal cancer (CRC) remains one of the most common and deadly malignancies worldwide, accounting for nearly 2 million new cases and almost 1 million deaths each year [1]. It consistently ranks among the top causes of cancer‐related mortality globally and in Taiwan, representing a major public health concern [2]. For CRC patients with locally advanced disease, neoadjuvant chemoradiotherapy (NACRT) followed by radical surgery is the standard of care, offering the potential for tumor downstaging, local control, and occasionally pathological complete response (pCR), which is associated with favorable prognosis [3]. However, treatment response to NACRT in CRC is highly heterogeneous, and a substantial proportion of patients derive limited benefit yet experience significant treatment‐related toxicity [4]. Accurately identifying responders and non‐responders in CRC before NACRT treatment is therefore essential to guide individualized treatment strategies and improve clinical outcomes.

Numerous clinical, pathological and molecular factors have been explored as potential predictors of response to NACRT in CRC. Tumor stage, histology, and baseline serum carcinoembryonic antigen (CEA) levels have been shown to influence treatment outcomes, with advanced stage, mucinous histology or elevated CEA levels associated with poor response [5, 6, 7], as do molecular alterations such as KRAS, TP53 and BRAF mutations along with systemic inflammation and immune microenvironmental status that also modulate radiosensitivity [8, 9]. In addition, circulating biomarkers including circulating tumor DNA (ctDNA) and plasma proteins (e.g., VEGFR3, EGFR, COX‐2) have been proposed as noninvasive predictors of NACRT efficacy [10, 11]. Beyond these established predictors, metabolic factors—particularly glycemic status—have also been proposed to influence treatment sensitivity. Clinical observations suggest that CRC patients with diabetes or elevated glucose levels might experience inferior outcomes [12, 13, 14]. Although experimental studies suggest that hyperglycemia could reduce radiosensitivity through mechanisms such as hypoxia‐inducible pathways or oxidative stress [15, 16], clinical evidence remains inconsistent and validated predictive models are lacking.

Radiomics has emerged as a powerful, noninvasive imaging approach that extracts quantitative features from routinely acquired medical scans to characterize tumor heterogeneity and biological behavior, thereby enabling individualized prediction of treatment response [17, 18, 19]. In CRC, radiomics has been increasingly applied in tumor detection, staging, and prediction of molecular characteristics, prognosis, and treatment response. Several studies have demonstrated the utility of computed tomography (CT)‐, magnetic resonance imaging (MRI)‐, and positron emission tomography (PET)/CT‐based radiomic features in differentiating benign from malignant lesions [20, 21], assessing lymph node metastasis [22], and identifying high‐risk disease phenotypes [23]. In addition, radiomics models have been developed to predict survival outcomes [24, 25], tumor recurrence [26], and therapeutic response to NACRT [24, 27], chemotherapy [28, 29] and immunotherapy [21]. Collectively, these studies highlight radiomics as a promising quantitative biomarker that can complement conventional imaging and provide deeper insight into the biological behavior of CRC. Before radiotherapy, patients routinely undergo CT simulation for localization. In this study, CT images obtained from CT simulation were used for CT‐based radiomics analysis.

Despite their variable predictive potential, clinical biomarkers and radiomics provide distinct but complementary information: the former reflecting systemic tumor burden or metabolic status, and the latter capturing local intratumoral heterogeneity. Their integration may therefore yield superior predictive performance compared with single‐modality models. Accordingly, this study aimed to identify which routinely collected clinical biomarkers could most effectively enhance a CT‐based radiomics model for predicting treatment response after NACRT. Candidate biomarkers included the widely used tumor marker CEA and metabolic indicators: fasting glucose and HbA1c. By evaluating these factors within an integrated radiomics–clinical framework, we sought to determine which variables provide independent and additive predictive value.

## 2 Methods

### 2.1 Study Population

This retrospective single‐center study was approved by the Institutional Review Board of Kaohsiung Medical University Chung‐Ho Memorial Hospital (KMUHIRB‐E(I)‐20240367), with informed consent waived due to its retrospective nature. Ninety‐two patients with histologically confirmed CRC who received NACRT followed by curative surgery between January 2015 and December 2023 were enrolled. Inclusion criteria were: (1) clinically confirmed stages II–III CRC according to the AJCC 8th edition; (2) completion of standard NACRT followed by radical resection with available postoperative pathology; (3) availability of pre‐treatment radiotherapy planning CT images of adequate quality for radiomics analysis; and (4) available pre‐NACRT laboratory data including CEA, fasting glucose, and HbA1c. Exclusion criteria included: (1) prior abdominal or pelvic radiotherapy or chemotherapy before NACRT; (2) presence of distant metastasis at diagnosis; (3) synchronous or metachronous malignancies; (4) inadequate image quality or incomplete tumor contour on planning CT; and (5) missing essential clinical or laboratory information.

### 2.2 Clinical and Pathological Data

Demographic and clinical characteristics, including age, sex, tumor location (colon or rectum), and pretreatment clinical stage, were collected from medical records. Laboratory parameters obtained within 2 weeks before NACRT included fasting glucose, HbA1c, and CEA levels. Pathologic data were extracted from postoperative reports and comprised tumor size, T and N stage, histological type, differentiation grade, lymphovascular invasion (LVI), perineural invasion (PNI), and tumor regression grade (TRG), with TRG being assessed using the modified Ryan scheme. For descriptive presentation in this study, TRG 0, 1, 2 and 3 were labeled as pathological complete response (pCR), partial response (PR), stable disease (SD), and progressive disease (PD) respectively; however, these labels represent study‐defined pathological response categories rather than Response Evaluation Criteria in Solid Tumor s‐based (RECIST) clinical response categories.

### 2.3 Image Acquisition, Segmentation, and CT‐Based Radiomics Feature Extraction

Pre‐treatment radiotherapy non‐contrast planning CT scans were acquired using a Philips Brilliance 16 CT simulator (Philips Medical Systems, The Netherlands) at the Department of Radiation Oncology, Kaohsiung Medical University Hospital, with the following parameters: tube voltage 120 kV, tube current 350 mAs, and slice thickness 5 mm. The gross tumor volume (GTV) was manually delineated on each axial slice by a radiation oncologist and confirmed by a senior specialist. To ensure subsequent image feature reproducibility and cross‐patient comparability, the images were resampled to a voxel size of 1 × 1 × 5 mm^3^, rescaled to −1000 to +3000 HU, and discretized into 400 gray levels with a fixed bin width of 10 HU. Three‐dimensional (3D) radiomic features were extracted from the GTV using LIFEx software (v25.06.1, www.lifexsoft.org). A total of 179 features were initially obtained; after exclusion of missing or low‐variance parameters, 111 were retained for subsequent analysis. These features encompassed morphological descriptors, first‐order intensity statistics, and texture metrics derived from gray‐level co‐occurrence matrix (GLCM), gray‐level run‐length matrix (GLRLM), neighborhood gray‐tone difference matrix (NGTDM), and gray‐level size‐zone matrix (GLSZM) matrices.

### 2.4 Feature Selection and Model Construction

The 92 patients were randomly divided into a derivation cohort (*n* = 65) and a validation cohort (*n* = 27) at a 7:3 ratio. Radiomic features were normalized to a range of 0–1 using min–max scaling before analysis. Feature selection was conducted in the derivation cohort using least absolute shrinkage and selection operator (LASSO) regression with 10‐fold cross‐validation to determine the optimal lambda (*λ*) that minimized the mean squared error (MSE). Features with non‐zero coefficients at the optimal *λ* were retained to construct the radiomics signature, and the radiomics score (Rad‐score) for each patient was calculated as a linear combination of the selected features weighted by the corresponding coefficients. In addition, univariate and multivariate logistic regression analyses were used to evaluate the clinical factors associated with NACRT treatment outcome (pCR vs. non‐pCR), and the variables with *p* < 0.1 were included in the multivariate model. A nomogram was then constructed based on the final multivariate model.

### 2.5 Model Evaluation and Statistical Analysis

The predictive performance of the model for pCR in both derivation and validation set were evaluated using receiver operating characteristic (ROC) analysis, and the area under ROC curve (AUC) were reported. Calibration test with bootstrap resampling was performed using the Hosmer–Lemeshow test and illustrated using calibration curves. *p* < 0.05 was considered statistically significant. All analyses were performed using R (v4.4.2; packages glmnet, pROC, rms).

## 3 Results

### 3.1 Baseline Characteristics of the Study Population

A total of 92 CRC patients who underwent NACRT between 2015 and 2023 were retrospectively analyzed. As shown in Table 1, the median age at diagnosis was 63 years, and 58 patients (63%) were male. Most cases were clinically staged as stages II or III according to the AJCC 8th edition. All patients completed NACRT and subsequently underwent curative resection, mainly through low anterior resection or abdominoperineal resection depending on tumor location. Post‐treatment pathological evaluation showed that 27 patients (29%) achieved pathological complete response (pCR, TRG 0), whereas the remaining patients exhibited varying degrees of residual tumor regression (TRG 1–3). For subsequent model construction and validation, the cohort was randomly divided into a derivation (training) set (*n* = 65) and a validation set (*n* = 27) at a 7:3 ratio, ensuring a balanced distribution of pCR cases between the two groups. There were no significant differences in pathological response, tumor characteristics, pathological parameters, or clinical biomarkers between the derivation and validation cohorts (all *p* > 0.05).

**TABLE 1:** Baseline characteristics of the study cohorts.

Characteristics	Overall	Derivation cohort	Validation cohort	*p*
Case #	92	65	27	
Gender, male	58 (63%)	42 (65%)	16 (59%)	
Diagnosis age, years	62.9 ± 11.4	63.4 ± 11.5	61.7 ± 11.3	0.725
Tumor characteristics				
Position				
Lower/middle	39 (42%)	28 (43%)	11 (41%)	
Upper	51 (55%)	35 (54%)	16 (59%)	
A‐colon	2 (2.2%)	2 (3.1%)	0 (0%)	
Clinical tumor staging				1.000
2	1 (1.1%)	1 (1.5%)	0 (0%)	
3	74 (80%)	52 (80%)	22 (81%)	
4	17 (18%)	12 (18%)	5 (19%)	
Clinical stage				0.679
II	18 (20%)	12 (18%)	6 (22%)	
III	74 (80%)	53 (82%)	21 (78%)	
Pathological response				0.230
pCR	27 (29%)	17 (26%)	10 (37%)	
PR	42 (46%)	33 (51%)	9 (33%)	
SD	20 (22%)	12 (18%)	8 (30%)	
PD	3 (3.3%)	3 (4.6%)	0 (0%)	
Pathological tumor staging				0.044
0	27 (29%)	17 (26%)	10 (37%)	
1	9 (9.8%)	8 (12%)	1 (3.7%)	
2	22 (24%)	19 (29%)	3 (11%)	
3	32 (35%)	21 (32%)	11 (41%)	
4	2 (2.2%)	0 (0%)	2 (7.4%)	
pLN+	17 (18%)	14 (22%)	3 (11%)	
Pathological stage				0.031
0	26 (28%)	16 (25%)	10 (37%)	
I	26 (28%)	22 (34%)	4 (15%)	
II	23 (25%)	13 (20%)	10 (37%)	
III	16 (17%)	14 (22%)	2 (7.4%)	
IV	1 (1.1%)	0 (0%)	1 (3.7%)	
LVI+	5 (5.4%)	4 (6.2%)	1 (3.7%)	1.000
PNI+	11 (12%)	7 (11%)	4 (15%)	0.725
Surgery				0.870
No OP	1 (1.1%)	1 (1.5%)	0 (0%)	
Traditional	56 (61%)	40 (62%)	16 (59%)	
Robotic	35 (38%)	24 (37%)	11 (41%)	
Clinical marker				
HbA1C	6.2 ± 1.3	6.1 ± 1.1	6.5 ± 1.8	0.823
Fasting glucose	124.7 ± 45.0	121.2 ± 44.8	133.0 ± 45.2	0.066
CEA, high	40 (43%)	27 (42%)	13 (48%)	0.560

### 3.2 CT‐Based Radiomics Feature Selection

**FIGURE 1:**
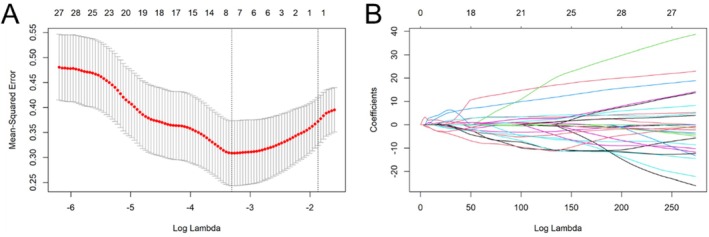
Radiomics feature selection using LASSO regression model. (A) Tuning parameter (*λ*) selection in the LASSO model via 10‐fold cross‐validation. The vertical dashed line indicates the optimal *λ* selected based on the minimum mean squared error (MSE). (B) LASSO coefficient profiles of the 111 candidate radiomics features as a function of Log Lambda.

**FIGURE 2:**
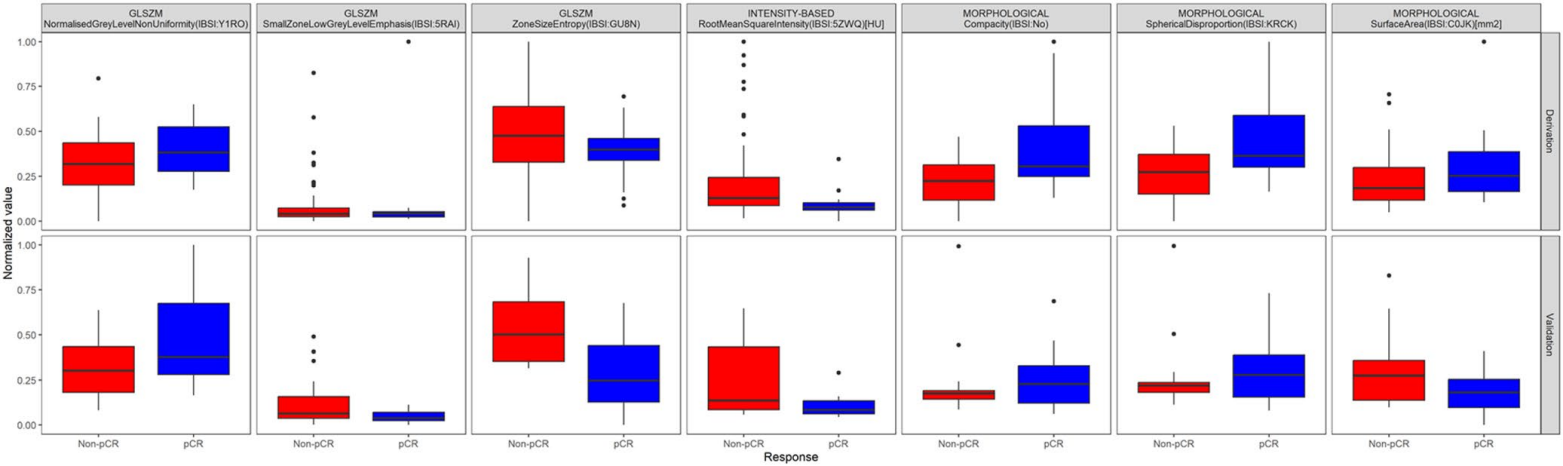
Distribution of the seven LASSO‐selected radiomic features. Box plots comparing the normalized values of the seven features retained by the LASSO model. Comparisons are shown between patients with pathological complete response (pCR, blue) and non‐pCR (red) in both derivation (top row) and validation (bottom row) cohorts.

A total of 111 radiomic features extracted from pre‐radiotherapy non‐contrast planning CT images were included in the feature selection process after excluding parameters with missing values or low variance. Least absolute shrinkage and selection operator (LASSO) logistic regression was applied within the derivation cohort to identify features most strongly associated with pCR. The tuning parameter *λ* was optimized through 10‐fold cross‐validation, with the minimum mean squared error used as the selection criterion. The cross‐validation curve and the coefficient profile path are shown in Figure 1, where seven features with non‐zero coefficients were retained for subsequent model construction. The selected features were derived from multiple categories, including first‐order intensity‐based and texture metrics from the GLCM, GLRLM, and GLSZM. Their normalized distributions and relative weights derived from the LASSO model are presented in Figure 2. Note that each selected feature alone showed limited discriminative power between pCR and non‐pCR groups in both the derivation and validation cohorts.

### 3.3 CT‐Based Radiomics Signature Performance

**FIGURE 3:**
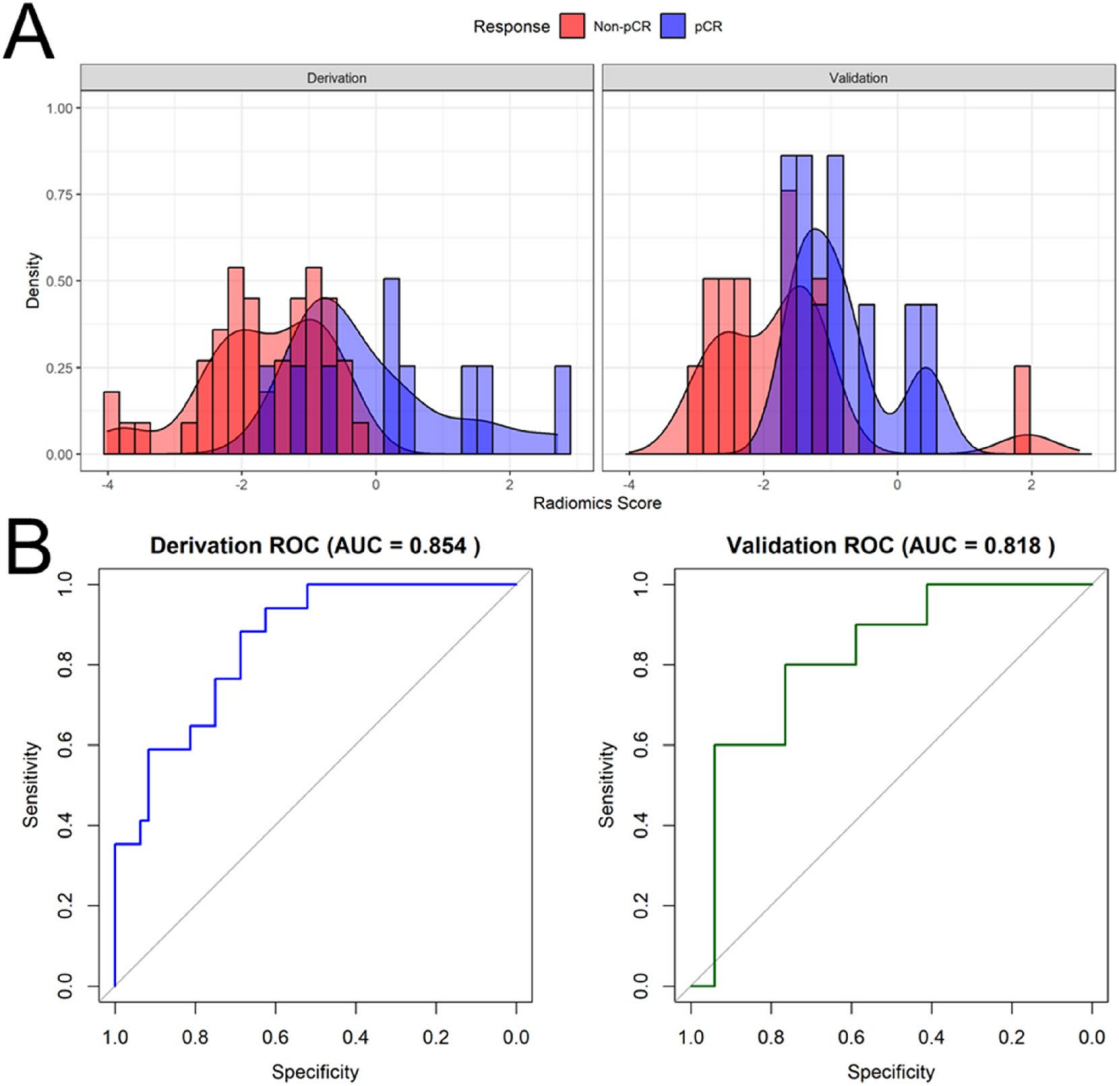
Performance of the radiomics signature (Rad‐score) in predicting pathological complete response (pCR). (A) Distribution (density plots and histograms) of the Rad‐score for patients with pCR (blue) and non‐pCR (red) in derivation and validation cohorts. (B) Receiver operating characteristic (ROC) curves illustrating the discriminative performance of the Rad‐score in derivation (area under the curve [AUC] = 0.854) and validation (AUC = 0.818) cohorts.

The seven selected radiomic features were linearly combined according to their respective LASSO coefficients to calculate a radiomics score (Rad‐score) for each patient. This Rad‐score served as a composite quantitative imaging biomarker summarizing the multi‐parametric information from the radiomics signature. The distribution of Rad‐scores between pCR and non‐pCR groups is illustrated in Figure 3A for both the derivation cohort and the validation cohort, showing higher overall Rad‐scores in patients who achieved pCR. The discriminative performance of the radiomics signature for identifying pCR was further assessed using ROC curve analysis (Figure 3B). The Rad‐score demonstrated good separation between the two response groups in both cohorts, confirming that the integrated radiomics signature could effectively distinguish patients likely to achieve pCR after NACRT. In contrast, conventional clinical parameters such as sex, age at diagnosis, tumor location and clinical stage did not significantly differ between patients with and without pCR (all *p* > 0.05). Only post‐treatment pathological variables such as tumor regression grade (TRG), pathological stage, LVI, PNI and lymph node status showed expected correlations with pCR, reflecting their direct association with postoperative pathology (Tables 2 and 3).

**TABLE 2:** Baseline characteristics of derivation set.

Characteristics	Derivation	Non‐pCR	pCR	*p*
Case no.	65	48	17	
Gender, male	42 (65%)	32 (67%)	10 (59%)	0.561
Diagnosis age, years	63.4 ± 11.5	62.7 ± 12.2	65.4 ± 9.2	0.382
Tumor characteristics				
Position				0.505
Lower/middle	28 (43%)	20 (42%)	8 (47%)	
Upper	35 (54%)	27 (56%)	8 (47%)	
A‐colon	2 (3.1%)	1 (2.1%)	1 (5.9%)	
Clinical tumor staging				0.174
2	1 (1.5%)	0 (0%)	1 (5.9%)	
3	52 (80%)	40 (83%)	12 (71%)	
4	12 (18%)	8 (17%)	4 (24%)	
Clinical stage				
II	53 (82%)	39 (81%)	14 (82%)	1.000
III	12 (18%)	9 (19%)	3 (18%)	1.000
Pathological response				< 0.001
pCR	17 (26%)	—	17 (100%)	
PR	33 (51%)	33 (69%)	—	
SD	12 (18%)	12 (25%)	—	
PD	3 (4.6%)	3 (6.3%)	—	
Pathological tumor staging	53 (82%)	39 (81%)	14 (82%)	
0				< 0.001
1	17 (26%)	0 (0%)	17 (100%)	
2	8 (12%)	8 (17%)	0 (0%)	
3	19 (29%)	19 (40%)	0 (0%)	
4	21 (32%)	21 (44%)	0 (0%)	
pLN+	14 (22%)	13 (27%)	1 (5.9%)	0.091
Pathological stage				< 0.001
0	16 (25%)	0 (0%)	16 (94%)	
I	22 (34%)	22 (46%)	0 (0%)	
II	13 (20%)	13 (27%)	0 (0%)	
III	14 (22%)	13 (27%)	1 (5.9%)	
IV	—	—	—	
LVI+	4 (6.2%)	4 (8.3%)	0 (0%)	0.566
PNI+	7 (11%)	7 (15%)	0 (0%)	0.176
Surgery				0.373
No OP	1 (1.5%)	0 (0%)	1 (5.9%)	
Traditional	40 (62%)	30 (63%)	10 (59%)	
Robotic	24 (37%)	18 (38%)	6 (35%)	
Clinical marker				
HbA1C	6.1 ± 1.1	6.1 ± 1.2	6.3 ± 0.9	0.081
Fasting glucose	121.2 ± 44.8	122.5 ± 49.4	117.7 ± 29.0	0.321
CEA, high	27 (42%)	24 (50%)	3 (18%)	0.020
Radiomics score	−1.3 ± 1.2	−1.7 ± 0.9	−0.1 ± 1.1	< 0.001

**TABLE 3:** Baseline characteristics of validation set.

Characteristics	Validation	Non‐pCR	pCR	*p*
Case no.	27	17	10	
Gender, male	16 (59%)	11 (65%)	5 (50%)	0.687
Diagnosis age, years	61.7 ± 11.3	61.0 ± 11.3	63.0 ± 11.7	0.392
Tumor characteristics				
Position				0.224
Lower/middle	11 (41%)	5 (29%)	6 (60%)	
Upper	16 (59%)	12 (71%)	4 (40%)	
A‐colon	—	—	—	
Clinical tumor staging				0.124
2	—	—	—	
3	22 (81%)	12 (71%)	10 (100%)	
4	5 (19%)	5 (29%)	0 (0%)	
Clinical stage				< 0.001
II	5 (19%)	5 (29%)	0 (0%)	
III	21 (78%)	17 (100%)	4 (40%)	
Pathological response				< 0.001
pCR	10 (37%)	0 (0%)	10 (100%)	
PR	9 (33%)	9 (53%)	0 (0%)	
SD	8 (30%)	8 (47%)	0 (0%)	
PD				
Pathological tumor staging				< 0.001
0	10 (37%)	0 (0%)	10 (100%)	
1	1 (3.7%)	1 (5.9%)	0 (0%)	
2	3 (11%)	3 (18%)	0 (0%)	
3	11 (41%)	11 (65%)	0 (0%)	
4	2 (7.4%)	2 (12%)	0 (0%)	
pLN+	3 (11%)	3 (18%)	0 (0%)	0.274
Pathological stage				< 0.001
0	10 (37%)	0 (0%)	10 (100%)	
I	4 (15%)	4 (24%)	0 (0%)	
II	10 (37%)	10 (59%)	0 (0%)	
III	2 (7.4%)	2 (12%)	0 (0%)	
IV	1 (3.7%)	1 (5.9%)	0 (0%)	
LVI+	1 (3.7%)	1 (5.9%)	0 (0%)	1.000
PNI+	4 (15%)	4 (24%)	0 (0%)	0.264
Surgery				0.687
No OP	—	—	—	
Traditional	16 (59%)	11 (65%)	5 (50%)	
Robotic	11 (41%)	6 (35%)	5 (50%)	
Clinical marker				
HbA1C	6.5 ± 1.8	6.4 ± 1.9	6.5 ± 1.5	0.801
Fasting glucose	133.0 ± 45.2	141.3 ± 52.4	119.0 ± 26.0	0.258
CEA, high	13 (48%)	11 (65%)	2 (20%)	0.046
Radiomics score	−1.4 ± 1.1	−1.7 ± 1.1	−0.8 ± 0.7	0.006

### 3.4 Identification of Independent Predictors for pCR

To further identify key clinical and radiomic factors capable of predicting pCR, both univariate and multivariate logistic regression analyses were performed in the derivation cohort (*n* = 65). In the univariate analysis, we assessed the predictive value of the Rad‐score alongside various clinical variables. Consistent with our preliminary findings, most baseline clinical characteristics—including sex, age at diagnosis, fasting glucose, and clinical stage—were not significantly associated with pCR status (all *p* > 0.05). Notably, only two variables showed a significant association with achieving pCR. A low pretreatment CEA level was a strong predictor (OR = 4.67; 95% CI: 1.32–22.20; *p* = 0.027), as was a higher Rad‐score (OR = 9.08; 95% CI: 3.02–47.10; *p* = 0.001). In the multivariate model constructed using a stepwise selection method, both a low CEA level (adjusted OR = 15.20; 95% CI: 2.05–289.00; *p* = 0.024) and a high Rad‐score (adjusted OR = 10.20; 95% CI: 3.14–68.80; *p* = 0.002) remained independent predictors of pCR. HbA1c showed a potential trend (*p* = 0.069) but did not reach statistical significance. The full regression results are summarized in Table 4. These findings suggest that combining the radiomics signature with the pretreatment CEA level provides complementary and independent predictive information for achieving pCR.

**TABLE 4:** Univariate and multivariate logistic regression analysis results for pCR agreement in the derivation cohort (*n* = 65).

Characteristics	Comparison	Univariate		Multivariate^a^	
		OR (95% CI)	*p*	OR (95% CI)	*p*
Gender, male	Male versus female	0.71 (0.23, 2.29)	0.562	—	
Diagnosis age	Years	1.02 (0.97, 1.07)	0.402	—	
HbA1c	Continuous	1.23 (0.75, 1.98)	0.397	2.22 (0.97, 5.81)	0.069
Fasting glucose	Continuous	0.997 (0.98, 1.01)	0.703	—	
Clinical tumor staging	4 versus 2/3	1.54 (0.36, 5.78)	0.533	—	
Clinical stage	III versus II	1.08 (0.27, 5.37)	0.920	—	
CEA^b^	Low versus high	4.67 (1.32, 22.20)	0.027	15.20 (2.05, 289.00)	**0.024**
Radiomics score^b^	Continuous	9.08 (3.02, 47.10)	0.001	10.20 (3.14, 68.80)	**0.002**

*Note:* Bold values indicates statistical significance *p* < 0.05.

^a^ The included variables in the multivariate model are selected via stepwise selection.

^b^ Variables with significant results in the multivariate model are selected to construct a final estimation model for pCR probability as shown in nomogram.

### 3.5 Development and Validation of a Predictive Nomogram

**FIGURE 4:**
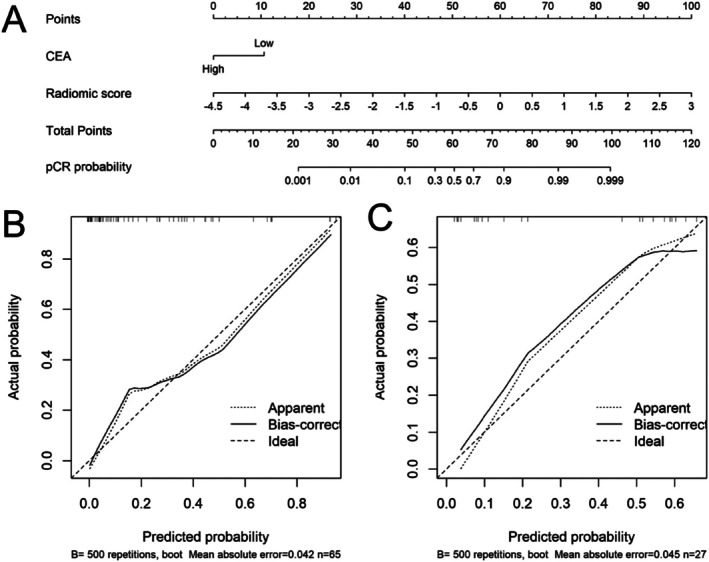
Development and validation of the nomogram for predicting pathological complete response (pCR). (A) The nomogram integrating the two independent predictors: Pretreatment carcinoembryonic antigen (CEA) level and the radiomics score (Rad‐score). (B) Calibration curve of the nomogram in the derivation cohort (*n* = 65), demonstrating model performance against an ideal prediction (dashed line). (C) Calibration curve of the nomogram in the internal validation cohort (*n* = 27).

Based on the independent predictors identified in the multivariate analysis, a nomogram was developed to provide a quantitative tool for individualized prediction of pCR probability. This model integrated the two most significant variables: pretreatment serum CEA level and the Rad‐score (Figure 4A). The integrated nomogram demonstrated excellent discriminative ability, achieving an AUC of 0.898 (95% CI: 0.823–0.974) in the derivation cohort, outperforming both the Rad‐score (AUC = 0.854) and CEA alone (AUC = 0.662). This performance was maintained in the validation cohort, with an AUC of 0.853 (95% CI: 0.705–1.000) for the integrated model, compared to 0.818 for the Rad‐score and 0.724 for CEA alone. Furthermore, the performance of the nomogram was evaluated using calibration curves, which demonstrated excellent agreement between predicted probabilities and actual observations. In the derivation cohort, the calibration curve closely aligned with the ideal 45‐degree line (MAE = 0.042; Figure 4B). The model was further assessed in the internal validation cohort, where the calibration curve also showed strong agreement between predicted and observed pCR probabilities (MAE = 0.045; Figure 4C). These findings confirm that the proposed nomogram was well calibrated and showed robust performance in both derivation and internal validation cohorts.

## 4 Discussion

This study sought to determine which routinely measured clinical biomarkers could provide additive predictive accuracy when integrated with a CT–based radiomics model for NACRT response. Although hyperglycemia indicators were included based on their biological rationale, the analysis ultimately showed that they did not provide additional predictive value; instead, the combination of radiomics and CEA yielded the strongest and most stable predictive performance, indicating complementary value between quantitative imaging heterogeneity and systemic tumor burden. The combined model demonstrated strong discrimination and excellent calibration in both derivation and validation cohorts, supporting the use of standard non‐contrast planning CT image radiomics together with CEA as a practical and biologically meaningful approach for improving NACRT response prediction.

Previous radiomics studies have mainly relied on contrast‐enhanced CT (CE‐CT) or MRI to assess treatment response in locally advanced rectal cancer. MRI‐based models incorporating T2‐weighted, diffusion‐weighted (DWI), or dynamic contrast‐enhanced (DCE) sequences have achieved high predictive performance, with AUCs often ranging from 0.78 to 0.95 [30, 31, 32]. MRI offers several advantages, including excellent soft‐tissue contrast and the ability of multi‐parametric imaging to capture structural and functional tumor characteristics, which might better reflect treatment‐induced changes in tumor cellularity and microcirculation. However, the clinical translation of MRI radiomics also has important limitations, including longer acquisition times, higher costs, susceptibility to motion artifacts such as bowel peristalsis, and insufficient standardization across scanners, field strengths, and sequence parameters, all of which could affect the reproducibility of radiomic features.

Similarly, CT‐based radiomics using contrast‐enhanced scans has been reported to predict treatment response with AUCs of 0.70–0.95 in validation cohorts [33, 34, 35]. CE‐CT is widely used in routine clinical staging and offers important advantages, particularly improved lesion conspicuity and the ability to reflect enhancement‐related tumor characteristics such as vascularity and perfusion, which might be relevant to tumor biology and radiosensitivity. However, CE‐CT‐based radiomics can also be influenced by variability in contrast phase, injection timing, and acquisition protocols that can affect feature reproducibility across patients and institutions.

In contrast, non‐contrast radiotherapy planning CT has both strengths and limitations. Its main limitation is the lack of enhancement information, which might reduce sensitivity to perfusion‐related tumor characteristics. Nevertheless, planning CT is mandatorily acquired for patients undergoing NACRT preparation and is obtained in the treatment‐position setting used for radiotherapy simulation. This provides a standardized image source that is directly integrated into the radiotherapy workflow thereby reducing positional inconsistency between imaging and treatment planning. In addition, non‐contrast imaging avoids the risks associated with intravenous contrast administration and can therefore be more broadly applicable in routine practice. Therefore, rather than suggesting that non‐contrast planning CT is superior to CE‐CT, our findings indicate that routinely acquired non‐contrast planning CT still contains meaningful information on tissue density and intratumoral heterogeneity. When combined with a systemic biomarker such as CEA, these features were sufficient to achieve robust predictive performance in our cohort, although direct comparison between CE‐CT‐based and planning CT‐based radiomics models would be valuable in future studies.

In addition to the CT‐based radiomics signature, our results confirmed the significant predictive value of pretreatment CEA. The baseline serum CEA level was not only a significant predictor in the univariate analysis (*p* = 0.027) but a strong independent predictor of pCR as well, in the final multivariate model (*p* = 0.024). This finding, which is consistent with previous reports linking low baseline CEA to improved NACRT outcomes [36, 37], highlights its individual predictive capability. Crucially, both CEA and the Rad‐score remained statistically significant in the multivariate analysis, indicating that they provide complementary and non‐redundant information by integrating systemic tumor burden with intratumoral imaging heterogeneity. Our results therefore show that integrating the CEA level alongside CT‐based radiomic features led to a more robust predictive model. This aligns with the recent trend that multimodal models combining clinical and imaging predictors can enhance model discrimination and generalizability [29, 38, 39].

Furthermore, our findings suggest that the integrated model provides added predictive value beyond conventional pretreatment clinicopathological assessment. In routine clinical practice, variables such as age, sex, tumor location, and clinical stage are commonly considered when estimating prognosis and guiding treatment decisions, although in our cohort, these conventional pretreatment clinicopathological features did not show significant predictive value for pCR, whereas pretreatment CEA emerged as the only robust clinical predictor. This indicates that a model based solely on routinely available pretreatment clinical variables might possess limited ability to discriminate treatment response.

By incorporating the CT‐based radiomics signature, the integrated model captures quantitative intratumoral heterogeneity that is not reflected by standard clinical staging alone. In the derivation cohort, the integrated model achieved the highest discriminatory performance, with an AUC of 0.898, compared with 0.854 for the radiomics score alone and 0.662 for CEA alone. A similar pattern was observed in the validation cohort, with AUCs of 0.853 for the integrated model, 0.818 for the radiomics score alone, and 0.724 for CEA alone. Together, these findings support the added value of integrating radiomics with CEA for pCR prediction in this cohort.

The inclusion of fasting glucose and HbA1c for evaluation as predictors was driven by previous evidence suggesting that hyperglycemia promotes tumor aggressiveness and resistance to chemoradiotherapy in CRC [12, 13, 14]. High‐glucose conditions have been shown to enhance tumor proliferation, angiogenesis, and DNA repair, thereby reducing radiosensitivity [15, 16, 40]. However, in our cohort, fasting glucose and HbA1c did not show significant predictive value in univariate analysis. Although HbA1c showed a borderline association in the multivariate model (*p* = 0.069), this finding should be interpreted cautiously given the limited sample size and the lack of significance in univariate testing. Therefore, our data do not support the inclusion of single‐time‐point glycemic indices as robust independent predictors of pCR in the current model. Future studies incorporating longitudinal metabolic measurements should better clarify the relationship between systemic glucose dysregulation and treatment response.

Our findings also have important clinical implications. By leveraging existing planning CT images of radiotherapy, our proposed model provides a practical and noninvasive tool for individualized prediction of pCR without the need for additional radiographic imaging. This framework could help clinicians identify CRC patients more likely to achieve pCR, providing more benefit for organ‐preserving strategies while distinguishing poor NACRT responders requiring alternative therapeutic approaches. Furthermore, the consistent calibration and reproducibility across the derivation and internal validation cohorts suggest that this approach is robust and could be feasible for implementation in routine radiotherapy workflows for decision support.

Several limitations of this study should be considered. First, this was a retrospective single‐center study with a relatively modest sample size, particularly in the validation cohort (*n* = 27), which might increase the risk of model instability and patient selection bias. At the same time, the single‐center design provided a relatively homogeneous clinical and imaging setting, with more consistent NACRT protocols, surgical management, and CT acquisition parameters, which could help reduce inter‐institutional variability during initial model development. Although internal validation procedures, including 10‐fold cross‐validation and bootstrap‐based calibration assessment, were applied to reduce overfitting, larger multi‐center prospective studies are still needed to confirm the robustness and external generalizability of this model. Second, although CEA is a widely accessible standard‐of‐care biomarker, our model did not incorporate other systemic inflammatory indices, such as the neutrophil‐to‐lymphocyte ratio, or more advanced molecular biomarkers, such as ctDNA or KRAS mutation status. Future studies integrating these biological parameters with CT‐based radiomics should further improve predictive performance.

In conclusion, integrating CT‐based radiomics with pretreatment CEA levels significantly improved the prediction accuracy for therapeutic response following NACRT. This CT‐based radiomics–CEA model is entirely based on routinely collected clinical and imaging data, making it highly feasible for routine clinical practice.

## Funding

This study was supported by grants from Kaohsiung Medical University Hospital (KMUH112‐2R70, KMUH113‐3R61, KMUH112‐M206, KMUH113‐M303, KMUH114‐4R76); Kaohsiung Medical University (KMU‐M112016); and the National Science and Technology Council (NSTC113‐2314‐B‐037‐065), Taiwan.

## Conflicts of Interest

The authors declare no conflicts of interest.

## Data Availability

The data that support the findings of this study are available on request from the corresponding author. The data are not publicly available due to privacy or ethical restrictions.
